# The Psychosocial Impact of Receiving Whole Genome and Whole Exome Sequencing Results in Adults: A Systematic Review

**DOI:** 10.1002/jgc4.70291

**Published:** 2026-09-24

**Authors:** Monica Albu, Andrada Ciucă, Ramona Moldovan

**Affiliations:** ^1^ Department of Psychology Babeş‐Bolyai University Cluj‐Napoca Romania; ^2^ Division of Evolution and Genomic Sciences, School of Biological Science University of Manchester Manchester UK; ^3^ Manchester Centre for Genomic Medicine, St Mary's Hospital, Manchester University Hospitals NHS Foundation Trust Manchester Academic Health Science Centre Manchester UK

**Keywords:** exome sequencing, genome sequencing, psychological impact, return of results, sharing results

## Abstract

The expanding use of whole genome sequencing (WGS) and whole exome sequencing (WES) underscores the need to better understand the psychosocial impact of receiving a broader range of potential results than those generated by other types of genetic testing. This systematic review aimed to provide a comprehensive assessment of the psychosocial impact of WGS and WES testing in adult populations. An electronic literature search was conducted to identify studies published up to July 2025. Quantitative and qualitative studies involving adult population, published in English, and examining the psychosocial impact of WES and WGS test results were included in the analysis. The analysis included 45 studies (27 quantitative, 11 qualitative, 7 mixed‐methods). Findings were organized into four categories: positive experiences, negative experiences, uncertainty and results sharing. Positive experiences were common, with participants reporting satisfaction from gaining greater insight into their health; negative experiences were also frequently reported, but were typically of low intensity. Feelings of uncertainty were less present among individuals who received genetic counseling. Sharing of test results was widespread, with participants demonstrating a good understanding of the implications for both themselves and others. this systematic review provides an overview of the existing literature exploring the psychosocial impact of receiving WGS and WES results in the adult population. Overall, the findings indicate that the benefits generally outweigh the potential risks of psychological distress, and that sharing of results is common. However, further qualitative and longitudinal research with larger and more diverse samples, as well as more nuanced and sensitive instruments, are needed to identify individual differences in responses that will help inform the development of appropriate models of care.

## Abstract (highlights)

**What Is Known About This Topic:**

Previous research shows that receiving WGS/WES results can elicit both positive and negative psychosocial responses in adults, including satisfaction from health insights, emotional distress, uncertainty, and considerations around sharing results with family.

**What This Paper Adds to the Topic:**

This systematic review demonstrates that positive experiences generally outweigh psychological harms, highlights the mitigating role of genetic counseling in reducing distress and uncertainty, and underscores the need for more nuanced, longitudinal research to inform models of genomic care.

## 1 Introduction

Whole Genome Sequencing (WGS) and Whole Exome Sequencing (WES) are relatively new technologies in routine medical practice and are increasingly used across a wide range of clinical, research and commercial settings. This growing use underscores the need for a better understanding of the potential psychosocial impact of receiving this type of genetic test results in adult population (Flinter 2013; Middleton et al. 2016).

Gaining an accurate and comprehensive understanding of the psychosocial impact of receiving WES/WGS results is essential for informing appropriate and tailored healthcare. WGS/WES are genetic tests designed to identify genetic variations involved in pathology or other aspects of health (Pasche and Absher 2011). WES/WGS facilitate the exploration of genetic variation, providing researchers and clinicians with efficient solutions both for identifying specific variants in a targeted region and for searching more broadly for potential causative variations by enabling the simultaneous analysis of a large number of genes. Unlike targeted genetic tests, which focus on specific genes or variants associated with a suspected condition, WES examines the protein‐coding regions of the genome, while WGS analyzes both coding and non‐coding regions, providing a more comprehensive assessment of genetic variation (Bagger et al. 2024). As WES/WGS provide the most comprehensive genetic information currently available at the individual level, the receipt of such results has raised concerns among healthcare providers regarding potential psychosocial harms, such as distress, psychological burdens (Rokoszak et al. 2024), or challenges in communicating results.

The increasing complexity of WES/WGS analysis and interpretation in clinical genetic testing requires ongoing monitoring and regular updates of guidelines to ensure the appropriate integration of the ethical, social, and legal considerations, as well as continued relevance and clinical utility of WGS/WES results (Richards et al. 2015; Austin‐Tse et al. 2022). Regardless of the testing context, results generally fall into three categories: positive (i.e., providing information relevant to a diagnosis or disease risk), negative (i.e., no clinically relevant genetic variants identified), or uncertain results (i.e., variants of uncertain significance (VUS), where the impact on health of the changes identified remains unclear) (Hoffman‐Andrews 2017). Depending on the objectives of testing, results may also be classified as primary or secondary (incidental) findings. Primary findings refer to genetic variants that are directly relevant to the diagnostic indication for which sequencing was performed, whereas secondary or incidental findings include variants unrelated to the original indication but identified in the course of sequencing (Richards et al. 2015).

Also, depending on the clinical indication for testing, genomic sequencing may identify diagnostic variants related to the presenting condition, carrier status for recessive disorders, predisposition to hereditary cancer syndromes, susceptibility to neurological or other genetic conditions. The significance and personal relevance of these findings may vary according to the individual's medical context and the reason for which testing was performed (Lewis et al. 2016; Skinner et al. 2017; Biesecker et al. 2018; Carrasco et al. 2023).

Communication of genetic test results encompasses multiple dimensions, including which results should be disclosed, when and how to best communicate them, and who should be responsible for communicating the information, as well as how individuals and their family members receive, understand, and appropriately use genetic information (Berg et al. 2011). The reporting of results is shaped by individual's preferences and must navigate ethical and practical complexities, which numerous laws and policies seek to address. In this context, Thorogood et al. (2019) analyzed laws and policies governing genomic sequencing across 20 countries and identified substantial variation of approaches to the return of results. This heterogeneity reflected differences in cultural perspectives on individual and family needs, variations in national healthcare systems, resource constraints within the research sector, limited sensitivity to distinctions between normative terms such as “must”, “should” and “can” when articulating obligations with potential legal implications, and the conflations of research and clinical contexts. Genetic healthcare professionals (e.g., genetic counselors) typically play a central role in translating genomic test results into clinical care. Evidence suggests that individuals who received genetic counseling after obtaining their results experienced significantly less confusion and better psychological adjustment than those who received their results through other means, such as online platforms (Umstead et al. 2020). The psychosocial impact of genetic testing encompasses both psychological responses to receiving results and other social factors related to the communication of those results, including whether, how and with whom results are shared, the role of results in informing reproductive choices, as well as the factors shaping these decisions. To date, much of the research in this area has focused on pediatric population (i.e., children, adolescents and their families) demonstrating that genetic testing can provide relief and empowerment by resolving diagnostic uncertainty, while also potentially increasing anxiety and distress, particularly in the context of uncertain or positive findings. Parental responses vary according to health literacy, socioeconomic factors and the availability of support, underscoring the need for sensitive communication strategies and ongoing psychosocial care for families (Biesecker et al. 2025; Dolling 2025). In contrast, considerably less is known about the psychosocial impact of disclosing WES/WGS results to adults. Robinson et al. (2019) conducted a meta‐analysis across diverse populations to examine psychological responses following the disclosure of WGS/WES results and demonstrated no clinically significant psychological impairment following communication of results but, instead, a broad range of positive responses. In adult populations, Mighton et al. (2021) systematically reviewed and synthesized the literature on psychological and clinical responses to receiving a VUS identified through multigene panel testing or genomic sequencing. Their findings showed that patients receiving VUS reported greater concern about the genetic test than those receiving negative results, but less concern than patients receiving positive results, alongside an increased likelihood of engaging in health‐related behavioral changes. With respect to social outcomes, existing evidence indicates that individuals frequently share WGS/WES results with caregivers and healthcare providers, suggesting a good understanding of their results and the potential implications they may have for others (Lewis et al. 2016).

As a result, the impact of genomic sequencing extends beyond its diagnostic utility and includes important psychosocial dimensions. In this review, psychosocial impact refers to the emotional, cognitive, behavioral, and communication‐related consequences associated with undergoing genomic testing and receiving genomic results. These may include anxiety, distress, uncertainty, relief, changes in risk perception, family communication, decision‐making, and adaptation to genetic information. Understanding these outcomes is essential for informing clinical practice and ensuring that genomic medicine is implemented in a patient‐centred manner.

This study aimed to systematically review and synthesize the quantitative and qualitative literature examining psychosocial responses following receipt of WGS and WES results, and to identify factors that shape psychological responses. The review sought to provide a nuanced and detailed overview, with particular emphasis on adults' self‐reported affective reactions. Understanding these responses is essential for informing the design and delivery of personalized healthcare to individuals undergoing complex genetic testing, for reasons that may include personal health and diagnostic goals (e.g., obtaining a diagnosis, guiding treatment and prevention strategies, or achieving peace of mind) (Biesecker et al. 2018; Peter et al. 2022; Abbott et al. 2025) as well as altruistic reasons (e.g., contributing to research) (Zierhut et al. 2015; Ormondroyd et al. 2020; Jamal et al. 2024). Recognizing these diverse motivations can support the tailoring of genetic counseling, management of expectations, validation of non‐diagnostic benefits, identification of socioeconomic barriers, and the use of positive emotional responses to support health‐related behavior change.

## 2 Methods

An extensive electronic literature search was conducted to identify the studies published until July 2025, with no predefined start date for the search. The PubMed, Scopus, and Web of Science databases were searched using keywords related to whole genome sequencing, whole exome sequencing, psychological and psychosocial impact, patient‐reported outcomes, and return of results. In the present review, psychosocial outcomes were conceptualized as encompassing emotional, cognitive, social, and behavioral responses associated with undergoing genomic testing and receiving genomic results. Behavioral outcomes were included as they represent important aspects of psychosocial adaptation, reflecting how individuals respond to, interpret, and act upon genomic information through healthcare engagement, decision‐making, risk‐management behaviors, and communication with family members and healthcare professionals. The complete search syntax is presented in Table 1. In addition, secondary sources, such as reviews or meta‐analyses, book chapters, and the reference lists of articles selected for full text assessment, were examined to identify additional studies.

**TABLE 1:** Search syntax.

(((((((((((((((((((((((((((“genome sequenc*” AND psychological) OR (“genome sequenc*” AND psychosocial)) OR (“genome sequenc*” AND emotion*)) OR (“genome sequenc*” AND distress)) OR (“genome sequenc*” AND wellbeing)) OR (“genome sequenc*” AND “patient‐reported outcomes”)) OR (“genome sequenc*” AND “return of results”)) OR (“genomic sequenc*” AND psychological)) OR (“genomic sequenc*” AND psychosocial)) OR (“genomic sequenc*” AND emotion*)) OR (“genomic sequenc*” AND distress)) OR (“genomic sequenc*” AND wellbeing)) OR (“genomic sequenc*” AND “patient‐reported outcomes”)) OR (“genomic sequenc*” AND “return of results”)) OR (“exome sequenc*” AND psychological)) OR (“exome sequenc*” AND psychosocial)) OR (“exome sequenc*” AND emotion*)) OR (“exome sequenc*” AND distress)) OR (“exome sequenc*” AND wellbeing)) OR (“exome sequenc*” AND “patient‐reported outcomes”)) OR (“exome sequenc*” AND “return of results”)) OR (“exomic sequenc*” AND psychological)) OR (“exomic sequenc*” AND psychosocial)) OR (“exomic sequenc*” AND emotion*)) OR (“exomic sequenc*” AND distress)) OR (“exomic sequenc*” AND wellbeing)) OR (“exomic sequenc*” AND “patient‐reported outcomes”)) OR (“exomic sequenc*” AND “return of results”)

Two reviewers (M.A. and A.C.) independently conducted all stages of the screening process, including title, abstract and full‐text review, using the same predefined inclusion and exclusion criteria throughout. Although no formal calibration or training exercise was conducted prior to screening, both reviewers followed the established eligibility criteria. Any discrepancies regarding study eligibility were discussed during consensus meetings and resolved through mutual agreement before proceeding to the subsequent stage of the review process.

The review was conducted and reported in accordance with the Preferred Reporting Items for Systematic Reviews and Meta‐Analyses guidelines (Page et al. 2021). The completed PRISMA 2020 Checklist is provided in the Supporting Information S1.

Studies were eligible for inclusion if they: (1) employed quantitative or qualitative methodologies; (2) were peer‐reviewed published in English; (3) explored the psychosocial impact of receiving WGS/WES test results; and (4) reported outcomes that were self‐reported by adult participants (i.e., ≥ 18 years). Studies that reported outcomes separately for adults and for children or relatives were included, provided that adult‐specific data could be extracted. Studies were excluded if they: (1) reported outcomes assessed or interpreted by experts, family members or the general public, rather than participants themselves; (2) involved prenatal, or children/adolescence testing contexts; (3) focused on the impact of testing on parents or caregivers; or (4) did not include sufficient data for analysis (i.e., studies reporting only pre‐test or post‐test assessments). Regarding study design, experimental, quasi‐experimental, correlational, observational, qualitative, and case studies were eligible for inclusion. Review articles, animal studies, conference abstracts, and opinion papers were excluded.

An exception to the exclusion criteria was made for the study by Rini et al. (2018), which included participants with an age range of 17–77, thereby extending 1 year below the predefined age limit. The study was included as the outcomes relevant to the aim of this study were assessed at 6–12 months after baseline, at which time the majority of participants were aged 18 years or older.

### 2.1 Data Extraction and Analysis

Data extraction was guided by a modified PICOS framework, including information on study characteristics (i.e., authors, year of publication, country), participants (i.e., sample size, gender, age, health condition), type of testing, outcomes, and study design (i.e., quantitative or qualitative). With respect to testing, we looked at whether WGS or WES was used; the provider of the results (e.g., genetics professionals, other clinical specialists or researchers); participants' motivation to undergo testing (e.g., clinical utility, curiosity, etc.); the type of result returned (e.g., primary or secondary findings; negative/positive results; pathogenic/probably pathogenic/not pathogenic/VUS results and others); and the time points of assessment (i.e., interval between receipt of results and assessment of psychological impact). Outcomes were categorized into affective (e.g., anxiety, depression), social (e.g., communication of results), and behavioral (e.g., behavioral lifestyle changes). Information on the instruments used to measure these constructs and other relevant outcomes were also extracted. Where feasible, extracted data were subjected to descriptive statistical analyses analyzed when possible (e.g., sums, means or percentages of relevant indicators).

Data were analyzed using a combination of thematic analysis and sentiment analysis see (Braun and Clarke 2006), with both approaches adapted to accommodate the mixed‐method nature of the studies. Thematic analysis was used to identify and synthesize recurring psychological themes (e.g., such as distress, relief, empowerment, uncertainty, and coping) across qualitative and quantitative findings, enabling exploration of the underlying meanings and experiences associated with receiving genomic results. Sentiment analysis complemented the thematic approach by focusing on the emotional valence of the participants' responses. For this, psychosocial outcomes and participant experiences reported across studies were extracted and grouped into broader sentiment themes. Recurring patterns related to emotional, cognitive, social, and behavioral responses to genomic sequencing were identified and compared across studies to develop an integrated understanding of the reported psychosocial impact. In quantitative studies, emotional responses were analyzed and categorized according to polarity (i.e., positive, negative or neutral) using a lexicon‐based method grounded in established dictionary meanings. In qualitative studies, text segments expressing subjective experiences were examined, and identified responses were similarly classified according to polarity, consistent with the approach used for quantitative data. For mixed‐methods studies, the quantitative component was included in the quantitative synthesis, whereas the qualitative component was included in the qualitative synthesis. Both types of evidence were subsequently integrated during the final narrative interpretation of the findings.

The methodological quality of the included studies was assessed using the Mixed Methods Appraisal Tool (Hong et al. 2018), which allows the evaluation of qualitative, quantitative, and mixed‐methods study designs within a single framework. Detailed quality assessment results are provided in the Supporting Information S2.

## 3 Results

**FIGURE 1:**
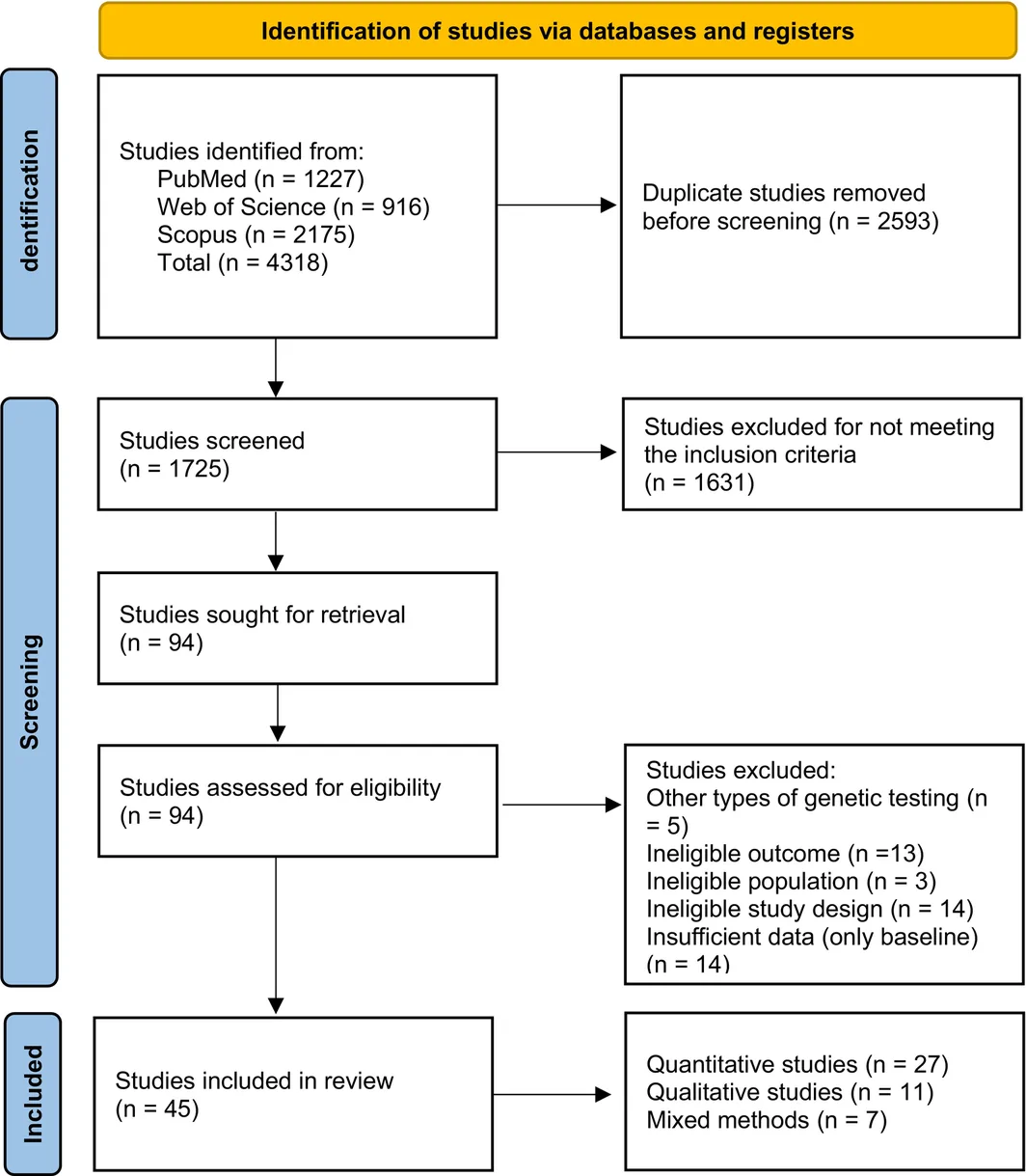
PRISMA flow diagram.

The search resulted in 4318 studies across the three databases, of which 2593 were identified as duplicates and removed. Abstracts extracted were managed using Zotero software (v6.0.36); duplicates were automatically identified and subsequently verified and excluded through manual review. The remaining 1725 studies were screened for title and abstract, resulting in the exclusion of 1631 studies according to the inclusion and exclusion criteria. A total of 94 studies were retrieved for full‐text review, of which 45 met the eligibility criteria and were included in the final analysis. Figure 1 shows the literature search and study selection flow diagram.

### 3.1 Characteristics of Included Studies

The total number of participants included across all studies was 7680; however, this figure includes overlapping participants, as several studies recruited individuals from the same cohort while measuring different outcomes. Table 2 presents the coding framework and key characteristics of the studies included in the review.

**TABLE 2:** Overview of the studies included in the review.

Study ID			Study design	Population				Intervention						Results		
No	Authors, publication year	Country		Sample size (*N*)	Demographics	Mean age/age range (years)	Health condition	Type of testing	Provider's background	Reason for testing	Results received	Intervention format	Assessment time point*	Assessed constructs	Instruments	Study results
1.	Abbasciano et al. (2022)	UK	Quantitative—Prospective and noninterventional feasibility study	70	41.4% (29) male 58.6% (41) female	48.5 (18–85)	NS‐TADs	WES	Genetic counselor or a multidisciplinary team (clinical geneticist, cardiac surgeon, bioinformatician)	Diagnostic purposes	VUS, findings in multiple genes	n.s.	3 months	Comprehension, Acceptability, Quality of Life, Anxiety and Depression	GAD‐7: PHQ‐9; QOLQ; SF‐36	No differences between baseline and 3‐month follow‐up scores for depression, anxiety, and self‐reported quality of life were observed.
2.	Beck et al. (2021)	USA	Qualitative—semi‐structured interviews	50	50% (25) male 50% (25) female	60.9 (42–71)	Healthy pop.	GS	Genetic counselor	Curiosity and health care purposes	Neutral results (benign and likely benign variants as well as VUS)	Online—mail	19–43 days	Reactions of receiving neutral results; Understanding the results package; Contextualizing the results; Misunderstandings about neutral results; Impact on health behaviors.	A semi‐structured, open‐ended interview guide administered by telephone	Individuals were satisfied with receiving neutral genomic sequencing results via email; this method did not cause anxiety or misunderstandings about health behaviors.
3.	Beil et al. (2021)	USA	Quantitative—observational	10	30% (3) male 70% (7) female	55 (19–64)	Aortic disease	WES	Genetic counselor	Medical utility	Pathogenic variants	In person	n.s.	Impact, understanding and satisfaction with the results disclosure process; Preferences and information sharing; Psychological impact; Decisional regret and satisfaction; Cost analysis.	Questionnaire designed for the study purpose; GCSS; FACToR; DRS; SWD.	Individuals manage the receive of their outcomes with low psychological distress and high levels of understanding.
4.	Biesecker et al. (2018)	USA	Quantitative—Randomized Clinical Trial	459	53.6% (246) male 46.4% (213) female	63.1	Healthy pop.	ES	Genetic counselor or through a web‐based platform	Research based	Carrier results	In person or online	1–6 months	Knowledge; Test‐specific distress; Decisional Conflict; Anxiety; Risk worry; Perceived risk; Communication of results to at‐risk relatives; Satisfaction.	Questionnaire designed for the study purpose; MICRA; DCS; STAI; GCSS; SWD.	The study found no indication of distress or other potential psychological harms that may arise from learning one's carrier status.
5.	Butterfield et al. (2019)	USA	Quantitative—correlational descriptive	131	33.6% (44) male 66.4% (87) female	18–61	Genetic conditions	GS	Clinical geneticist and/or genetic counselor	Diagnostic purposes	Negative results	By phone	1 week	Perceived risk; Nuances in understanding; Decisional regret; Support, conflict and family interactions; Health behaviors.	Questionnaire designed for the study purpose; MICRA.	Participants expressed no decisional regret and reported no intention to change health‐related behaviors.
6.	Carrasco et al. (2023)	Spain	Quantitative—Randomized Clinical Trial	32	43.8% (14) male 56.2% (18) female	46.5	Rare disorders	ES	n.s.	Diagnostic purposes	Secondary findings and incidental findings (pathogenic variants in cancer susceptibility genes)	n.s.	3 months	Psychological impact: distress, uncertainty, positive experiences.	MICRA	Participants who receive SF in cancer susceptibility genes from ES show considerably more distress and uncertainty compared to those who seek genetic testing due to a family history of cancer.
7.	Cheema et al. (2021)	USA	Qualitative—semi‐structured interviews	50	32% (16) male 68% (34) female	48 (20–73)	Healthy pop.	GS	Medical geneticist	To learn more about their health	Neutral results	By mail (with a follow‐up tele‐phone call) or in person	n.s.	Motivations for Pursuing GS; Concerns; Assessments of the value of GS.	Semi‐structured interview guide	Participants viewed the results as valuable and had minor concerns about the emotional burdens associated with receiving a positive result.
8.	Christensen et al. (2021)	USA	Quantitative—Randomized Clinical Trial	99	n.s.	18.7–84.6	Primary care and cardiology patients	GS	Participants' physician and cardiologists	Diagnostic and health care purposes	Findings related to the diagnosis of cardiomyopathy; VUS; secondary findings; carrier status.	n.s.	Immediately post disclosure, 6 weeks, 6 months	Health behaviors (doctor's recommendations, changes in health behaviors), psychological impact (anxiety, depression, impact of testing, affective responses).	Questionnaire designed for the study purpose and items adapted from multiple studies; HADS; MICRA.	GS does not cause distress to participants and shows an indirect impact on health behaviors by eliciting positive psychological responses.
9.	Ormondroyd et al. (2020)	UK	Qualitative—semi‐structured interviews	11	18.2% (2) male 81.8% (9) female	39–70	Healthy pop.	GS	n.s.	Altruistic reasons and personal and family reasons	Carrier results; negative results	Letter	n.s.	Understanding of genetic health risks; Motivation for and satisfaction; Personal consequences of participation in study; Family communication and outcomes; Responses of heath care system	Semi‐structured interview guide	Participants reported no ongoing anxiety caused by SF and followed the recommended health behaviors. In addition, they demonstrated understanding of genetic health risks and valued the information provided by the study.
10.	Gillman et al. (2022)	USA	Quantitative—longitudinal prospective	461	54% (248) male 46% (213) female	63.9	Heathy pop.	GS	Genetic counselor and/or a web‐based platform	Personal and family reasons	Carrier results—pathogenic or likely pathogenic variants, VUS	n.s.	Immediately post disclosure, 1 and 6 months after	Anticipated and Anticipatory Affects; State Anxiety; Decisional conflict; Distress; Uncertainty; Positive experiences; Communication of the results with the family; Intentions.	Questionnaire designed for the study purpose; STAI; Decisional Conflict Scale; MICRA	The study shows that future‐oriented emotions can play an important role in decisions that span the long term and such emotions bring many practical implications.
11.	Griesemer et al. (2019)	USA	Quantitative—descriptive	192	27.1% (52) male 72.9% (140) female	48.9/18–84	Physical health conditions of suspected genetic etiology	ES	Medical geneticist and genetic counselor	Personal and familial health implications	Positive, negative and uncertain results	In person	2 weeks – 6 months	Clinical variables (physical functioning, generalized distress); the type of hope and hope fulfillment; psychological adaptation (coping efficacy, self‐esteem, social integration, spiritual/existential well‐being).	Adapted version of Karnofsky Performance Status Scale; Questionnaire designed for the study purpose; PAS; HADS.	Psychological adaptation to new information in the context of genomic sequencing depends on patients' hopes and whether they have been fulfilled to some degree.
12.	Hart et al. (2019)	USA	Qualitative—semi‐structured interviews	10	n.s.	n.s.	Physical health conditions of genetic etiology	GS or ES	Medical geneticist, genetic counselor, specialist	Diagnostic purposes	Secondary findings	n.s.	< 6 months	Health services use; Insurance status; Psychological responses to the information.	Semi‐structured interview guide	Adults who received a SF with a significant health risk reflected on their current circumstances as well in pursuing follow‐up actions. However they were not anxious or distressed related to their SF.
13.	Jamal et al. (2024)	USA	Qualitative—cross‐sectional interviews	17	47.1% (8) male 52.9% (9) female	20–70+	Immune system conditions	WES	None	Health care and altruistic purposes	Uninformative results	Email	n.s.	Recall of informed consent; Views about uninformative results; Participants' causal attributions; Perceptions of residual disease risk; Screening behaviors and behavioral intentions to screen for additional diseases; Confusion and misconceptions; Questions and suggestions; Expectations regarding the future	Interview guide developed by teams members	Most participants did not remember the details of the informed consent process, had difficulties understanding what the testing had covered and they saw the uninformative results either as mundane or as unique.
14.	Lawal et al. (2018)	USA	Mixed methods—experimental	81	51.9% (42) male 48.1% (39) female	50–75	Coronary artery disease and healthy adults	ES	Researchers	Health care and altruistic purposes	VUS‐high or VUS‐low	In person	2 weeks	Behavioral intentions; Perceived risk, Perceived severity; Perceived value of Information; Self‐efficacy; Decision regret.	The behavioral intentions scale; adapted Champions' Perceived Severity Scale; DRS; questionnaire adapted from the literature or designed for the purpose of the study.	Participants were satisfied with the experience of results disclosure and understood the meaning of a genetic VUS result. The regret was higher in those who received a VUS‐low result which shows that participants were able to recognize a meaningful difference in the two sub‐classes of results.
15.	Lewis et al. (2016)	USA	Mixed methods—exploratory	29	52% (15) male 48% (14) female	52–71	Healthy pop.	ES	Medical geneticist and genetic counselor	Personal and familial health implications	Positive results—actionable sequencing results	In person or via telephone	3 months – 4 years	Medical and affective impact of sequence results; Personal utility of results: Whether and how results were shared within families; Preferences for receiving future results.	Semi‐structured interview guide; A modified version of MICRA.	The study shows neutral or positive emotional responses following receiving the results and a low level of distress in most participants. In addition, it shows that participants understand that the results have implications for others.
16.	Lewis et al. (2018)	USA	Quantitative—Randomized Controlled Trial	459	53.6% (246) male 46.4% (213) female	n.s. (post‐reproductive)	Healthy pop.	ES	Genetic counselor	Volunteer to participate	Carrier results	In person	Immediately post disclosure, 1 and 6 months after	Risk worry; Test‐related positive experiences; Attitudes; Decisional conflict; Satisfaction; Preferences; The value of counseling.	Questionnaire designed for the purpose of the study; a subscale from MICRA; DCS; SWD.	Participants who received counseling were more satisfied than those who did not, because it provided validation of their reactions and an opportunity for interpersonal interaction. Nevertheless, web‐based tools provide empiric evidence that counseling is not critical.
17.	Miller et al. (2019)	USA	Quantitative—descriptive	68	51.5% (35) male 48.5% (33) female	n.s.	Healthy pop.	ES	n.s.	Health care and altruistic purposes	VUS‐high or VUS‐low	In person	2 weeks	Health behaviors; Disclosure of VUS result; Psychological impact; Decisional conflict; Perceived value.	Modified version of MICRA; DCS; Questionnaire designed for the purpose of the study.	Participants who received VUS results showed minimal psychological distress and made few changes in health behaviors.
18.	Niu et al. (2019)	USA	Quantitative—randomized controlled trial	95	53.7% (51) male 46.3% (44) female	53.4	Inherited colorectal cancer/polyposis	ES	Medical geneticist and genetic counselor	Health care purposes	Medically actionable SFs, carrier status results and pharmacogenetic variants	In person	2 weeks and 4, 7, 10 months	Health care resource utilization; Psychological outcomes.	Questionnaire designed for the purpose of the study; FACToR, VR‐12; GAD‐7; PHQ‐9; MHI‐5.	The study did not observe differences in psychosocial impacts, healthcare use or family communication outcomes between the usual care arm and clinical exome sequencing arm.
19.	Peter et al. (2022)	UK	Mixed methods—a survey and in‐depth interviews	33	n.s.	n.s. (adults)	Genetically undiagnosed rare disease	GS	n.s.	Diagnostic and altruistic purposes	Positive results	n.s.	n.s.	Decision regret; Psychological impact; Motivations; Decision making; Communication of results; Experience of receiving GS results; Understanding and impact of GS results.	DRS; MICRA; Interview guide designed for the purpose of the study.	Receiving a diagnosis reveals minimal evidence of negative psychological impacts, supporting interview findings of both positive and negative emotional and psychological impacts irrespective of a genetic diagnosis.
20.	Reid et al. (2020)	USA	Quantitative—exploratory	312	56% (175) male 44% (137) female	60.1	Heterozygous carriers of at least one genetic variant.	ES	Genetic counselor or through a web‐based platform	Diagnostic and altruistic purposes	Carrier status results	In person or online	Immediately post disclosure, 1 and 6 months after	Attitudes; Injunctive norms; Decisional conflict; Disclosure.	DCS; Questionnaire designed for the purpose of the study.	The study shows that reactions to sequencing may be less positive when psychological and social factors, such as attitudes and injunctive norms, are not fully aligned with the decision to receive sequencing results.
21.	Rini et al. (2018)	USA	Quantitative—correlational	152	28.9% (44) male 71.1% (108) female	47.2	A suspected genetic disorder	ES	Medical geneticist and genetic counselor	Diagnostic purposes	Secondary findings	In person	6–12 months	Physical functioning; General health literacy; Anticipated regret; Intention to learn SFs.	Karnofsky Performance Status; 66‐item Rapid Estimate of Adult Literacy Measure; Questionnaire designed for the purpose of the study.	The study suggests that the intention to request SF with low or no medically actionability may be strongly influenced by participants' desire to avoid regret.
22.	Rini et al. (2019)	USA	Quantitative—longitudinal	199	27.1% (54) male 72.9% (145) female	50.8	Symptoms of an undiagnosed condition	ES	Medical geneticist and genetic counselor	Diagnostic purposes	Positive, uncertain, or negative diagnostic results	In person	2 weeks	Genomic knowledge; Health literacy; English proficiency; Test‐related distress and uncertainty; Decision regret; Perceived understanding.	UNC‐GKS; 66‐item Rapid Estimate of Adult Literacy Measure; 3‐item subscale of the Cultural Identity Scale; MICRA; DRS; Questionnaire designed for the purpose of the study.	The study reveals that general genomic knowledge can provide a foundation for better psychological sequencing outcomes.
23.	Rini et al. (2021)	USA	Quantitative—randomized controlled trial	335	32% (107) male 68% (228) female	Aprox 48	A health condition with a suspected genetic cause	ES	Medical geneticist and genetic counselor	Diagnostic purposes	Negative, un‐certain, or positive results and medically actionable secondary findings.	In person	2 weeks, 3 and 6 months	Physical functioning; Test‐related distress, positive experiences, and uncertainty; Generalized anxiety and depressive symptoms; Decision regret; Information seeking difficulty; Healthcare utilization.	Self‐report version of the Karnofsky scale; MICRA; HADS; DRS; 5 items adapted from HINTS; NHANES.	Participants did not appear to experience a negative effect that could indicate choice overload; they demonstrated limited short‐term benefits but no lasting benefit or harm.
24.	Roberts et al. (2018)	USA	Quantitative—prospective	295	50.2% (148) male 49.8% (147) female	59.1	Cancer	WES	The referring oncologist	Health care and altruistic purposes	Actionable findings	n.s.	2 weeks	Motivations and concerns regarding study participation; Knowledge; Expectations of study benefits; Decisional satisfaction	Questionnaire items created specifically for this study; 2 item from DRS; SWD.	Participants expected a wide range of benefits from participating in the study, which in most cases were not achieved, resulting in a relatively low level of decisional regret.
25.	Roche et al. (2019)	USA	Quantitative—correlational	155	25% (39) male 75% (116) female	47.3	Suspected genetic disorders	ES	Medical geneticist and certified genetic counselor	Diagnostic purposes	Non–medically actionable secondary findings	In person or by telephone	n.s.	Physical functioning; Generalized distress; General health literacy; Anticipated regret for not learning and for learning each NMASF category; Intention to learn NMASF; SF knowledge.	Self‐report version of the Karnofsky Performance Status scale; HADS; REALM; Questionnaire designed for the purpose of the study.	The study shows that factors that influence patients' preferences for genomic information that has little to no actionability have cognitive and emotional components.
26.	Sanderson et al. (2015)	USA	Mixed methods—observational and interview	19	52.6% (10) male 47.4% (9) female	n.s.	Healthy pop.	WGS	Students	Academic purpose	Carrier status result, VUS.	In person	Immediately after; 6 months	Educational outcomes (knowledge, attitudes, understanding of GS information); Depression; Anxiety; Psychological impact; Decision regret; Satisfaction with decision; Motivation; Understanding; Emotional impact.	Questionnaire designed for the purpose of the study; CES‐D; STAI; MICRA; DRS; SWD; In‐depth interviews.	Giving students the option of analyzing their own genomes may increase motivation to learn, but some students may experience personal WGS results–related distress and regret.
27.	Sanderson et al. (2017)	USA	Mixed methods—exploratory longitudinal	29	58.6% (17) male 41.4% (12) female	48.6	Healthy pop.	WGS	Medical geneticist and certified genetic counselor	Health care and altruistic purposes	Sequencing quality; ancestry; physical traits; pharmacogenomics; common polygenic disease risk; Alzheimer's disease risk; monogenic disease carrier variants and monogenic disease variants.	In person	Immediately after; 1 week; 6 months.	Reactions to personal results; understanding and recall of results; Satisfaction with procedures and materials; Depression; Anxiety; Quality of life; Positive experiences; Test‐related distress; Lifestyle behaviors.	Questionnaire designed for the purpose of the study; CES‐D; STAI; IQOLA; MICRA; SF‐36; BRFSS; Interview guide.	Most of participants reported positive reactions to receiving their results, but there were notable exceptions to this (e.g., participants whose likely pathogenic variants were reclassified as VUS or participants for whom their results and follow‐up were ambiguous).
28.	Sapp et al. (2018)	USA	Quantitaive ‐exploratory	92	48% (45) male 51% (47) female	50	A mix of clinical and research patients	ES	Genetic counselor	Diagnostic and altruistic purposes	Positive and negative secondary findings	By mail or telephone	4 months	Knowledge of sequencing; Perceived benefits of receiving SF; Depressive symptoms; Perceived physical, mental, and general health; Dispositional optimism; Perceptions of the meaning, value, and utility of receiving SF; Expectations and feelings of disappointment or reassurance.	KOGS; 4‐item scale (perceived benefits of receiving SF); CES‐D‐20; PROMIS‐Global Health; 3‐item version of LOT‐R; 5 open questions designed for the purpose of the study.	The study illustrated the feasibility of detecting and returning negative sequencing results to participants by telephone, with a low level of distress detected on their part.
29.	Shaibi et al. (2020)	USA	Qualitative—descriptive	500	24.4% (122) male 75.6 (378) female	49	Healthy pop.	Targeted GS	Researchers or medical geneticist	n.s.	Actionable Finding (pathogenic or likely pathogenic)	By mail or in person	1 week – 1 month	Challenges in returning research results and lessons learned.	Observations	The study shows that low health literacy may impact interpretation of results. For this reason, education about the test is very helpful in understanding the results and following health recommendations.
30.	Sie et al. (2015)	The Netherlands	Quantitative ‐observational prospective	111	50.4% (56) male 49.6% (55) female	49	Early‐onset colorectal/kidney cancer, deafness, blindness or movement disorder	ES	Medical geneticist	Diagnostic purposes	Pathogenic or possibly pathogenic mutations	n.s.	4 weeks	Satisfaction and regret; Heredity‐specific distress; Perceptions of hereditary and non‐genetic aetiologies; Expectation of unsolicited findings; Coping style; Quality of life; General psychological impact; Illness perception; The impact of genetic testing; Decision making and reactions.	Questionnaire designed for the purpose of the study; IES; TMSI; EORTC‐QLQ‐C30; GHQ‐12; B‐IPQ; MIHRA; Open‐ended questions.	The study reports increased satisfaction and decreased distress in most participants following receipt of sequencing results.
31.	Skinner et al. (2016)	USA	Qualitative—ethnographic observations and semi‐structured interview	20	25% (5) male 75% (15) female	47/18–66	Cancer. cardio‐genetics, neuromuscular problems and other conditions, including ophthalmology and mitochondrial.	ES	Clinician‐researchers	Diagnostic purposes	Negative results	In person	4 weeks	Experiences in the NCGENES study, including understandings of what was communicated in the return of results clinic session, and how they came to make sense of and value the negative result.	Interview questions designed for the purpose of this study.	The study shows that clinicians and patients construct the meaning of a negative result in ways that are uncertain, contingent, and multivalent; but invested with optimism, promise, and potentiality.
32.	Skinner et al. (2017)	USA	Qualitative—ethnographic observations and semi‐structured interviews	21	42.9% (9) male 57.1% (12) female	50/19–84	Patients suspected to have an underlying genetic etiology	ES	Clinical geneticists and genetic counselors	Diagnostic purposes	Uncertain diagnostic results	In person	4 weeks and 1 year	Understandings of their uncertain result; Concerns or psychosocial responses; Plans of action or accounts of actions they had taken based on the result (further testing or consultations).	Interview questions designed for the purpose of this study.	Participants understood their uncertain results in congruent ways with medical communication. They did not express regret for having learned the uncertain result; most regarded it as potentially valuable in the future.
33.	Smith et al. (2022)	USA	Qualitative—semi‐structured interviews	20	n.s.	18–54	Hereditary cancer and healthy adults	GS	n.s.	Diagnostic and health care purposes	Primary diagnostic results, secondary results, heterozygote status, VUS or inconclusive results and negative results.	n.s.	5 month (1 month – 5 years)	Clinical, emotional, behavioral, cognitive and social utility	A semi‐structured interview guide designed for the purpose of this study	Participants stated that GS results facilitate enhanced social support from the family but also from in‐person or online support groups for rare diseases. Positive responses were more common than negative ones, but among the negative ones was frustration, helplessness and fearfulness.
34.	Stuttgen et al. (2020)	USA	Quantitative—survey	1418	42.4% (601) male 57.6% (817) female	60.8/27–71	Healthy pop.	GS	None	Health care purposes	Negative screening diagnostic results	Mail	1–2 months	Patient interest in receiving negative genomic screening results; Patient reactions to receiving negative genomic screening results by mail; Patient perceived understanding of results.	82‐item survey consisting mostly of de novo items developed by the research team	Most participants value receiving negative GS results and are comfortable receiving such results by mail. However, it's not always the optimal solution, giving the fact that a great number of participants reported difficulty understanding some aspect of their GS results or were left with questions.
35.	Taber et al. (2018)	USA	Mixed method—experimental longitudinal	58	62% (36) male 38% (22) female	60	Myeloperoxidase deficiency, hemochromatosis, coronary artery disease or with other positive results from GS.	GS	Genetic counselor	Contacted for the reinterpretation of a gene variant	A carrier of Duarte galactosemia reinterpreted as positive result (still carrier) or negative result; carrier results with no reclassification.	In person or through a web platform	Immediately after disclosure—3 months	Recall, reactions, and thoughts about genetic information; Intention to share results; Perceived ambiguity; Intentions to learn genetic test results for preventable and unpreventable disease; Perceived utility; Negative emotions; Perceived accuracy.	Questionnaire designed for the purpose of the study; 3 items from MICRA.	The study indicates that participants understand reinterpretations that confer both neutral and major change. In the case of changes in low‐risk conditions, the responses of the participants indicated a low distress.
36.	Umstead et al. (2020)	USA	Mixed methods—Randomized controlled trial	462	53.6% (246) male 46.4% (213) female	63	Carriers of a variant of a gene associated with an autosomal recessive inherited disorder	ES	Genetic counselor or through a we platform	Health care purposes	Carrier status—pathogenic variants, probably pathogenic or zero pathogenic, VUS or zero VUS	In person or through a web platform	1–6 months	Perceived uncertainty (clinical, affective, evaluative); Anticipated ambiguity of sequencing results; Test‐related distress; Communication of results.	PUGS; the distress subscale of MICRA; Questionnaire designed for the purpose of the study	Disclosing results online may lead to greater uncertainty about health implications; the study additionally nuances the various uncertainties inherent in receiving sequencing results and their role.
37.	Schoot et al. (2021)	The Netherlands	Qualitative—semi‐structured interviews	10	n.s.	18–70	Oncological or cardiac diseases	ES	Clinical geneticists, counselor	Reproduction and understanding of the condition	Unsolicited findings predisposing to either oncological or cardiac disease.	n.s.	1–2 years	Psychological, physical, and financial impact; Actionability; Understanding; Pre‐test health; Social context.	A semi‐structured interview guide designed for the purpose of this study	Most participants expressed considerable psychological impact at the time of disclosure; however, patients see the testing as useful and would repeat it.
38.	Vassy et al. (2017)	USA	Quantitative—randomized trial	50	44% (22) male 56% (28) female	55/41–66	Heathy pop.	WGS	Primary care physicians	Health care purposes	Monogenic disease risk Results, carrier variants, pharmacogenomic associations, and polygenic risk estimates for cardiometabolic traits.	In person	Immediately after, 6 months	Healthcare utilization; Anxiety; Depression; Perceived health; Health behaviors; Molecular and clinical diagnoses; Appropriateness of clinical management; Healthcare costs.	HADS; Questionnaire designed for the purpose of the study.	The study shows that WGS neither worsened nor improved self‐rated health, anxiety, or depression scores among patients who underwent the testing compared to those who only had their family history observed.
39.	Wynn et al. (2018)	USA	Quantitative—longitudinal	107	31.8 (34) male 68.2 (73) female	50	Several condition with genetic etiology	ES	Genetic counselor	Health care and familial purposes	Secondary findings—results associated with an increased personal disease risk; carrier results, pharmacogenetics or reduced or average risk for Alzheimer's disease.	In person or by video conference	1 and 12 months	Expectancies regarding locus of control; Anxiety; Depression; Sleep quality; General genetic knowledge; Genetic stigma; Genetic secrecy, Healthy behavior; Health worry Social supports; Life changes; Psychological impact; Satisfaction.	HLC; BAI; PHQ–9; a question from the GSDS; questions from the Genetic Knowledge Measure; Questionnaire designed for the purpose of the study; MICRA; SWD; brief COPE scale.	The study shows that receiving secondary findings has little impact on most participants and the changes in psychosocial measures from baseline to post disclosure are imperceptible.
40.	Zierhut et al. (2015)	USA—Minnesota	Quantitative—survey	30	36.7 (11) male 63.3% (19) female	< 40 to > 60	Healthy pop.	WGS	A healthcare worker (primary care physicians, genetic counselors, and medical geneticists)	Professional enhancement, curiosity and personal health benefits	Medically actionable findings, carrier results, VUS.	In‐person in a clinical setting, by mail or e‐mail, or by phone.	3 months	Motivations; Aspirations; Concerns; Decision‐making; Communication of information; Anticipated reactions; Initial reactions; Information gained; Meaning of the results both personally and professionally; Regret; How WGS was affecting participants' current personal health and professional work.	DRS; Questionnaire designed for the purpose of the study.	Participants stated a greater impact on their professional life than on their health. Among the benefits of testing are the discovery of medically actionable findings, but they are not without risks, such as confusion, surprise and need for support. Over time, the
41.	Zoltick et al. (2019)	USA—Massachusetts	Quantitative—prospective longitudinal	543	61.4% (326) male 38.0% (202) female 0.6% (3) others	53	Healthy pop.	GS or ES	Optional appointment with a healthcare provider	Curiosity, familial and health care purposes	Monogenic disease findings in numerous genes	Online platform	3.8–8 months	Motivations and concerns; Psychological impact (psychological states and decisional regret); Perceived risk, utility and harm; Behavioral and medical responses; Genome sequencing knowledge.	Questionnaire designed for the purpose of the study; PHQ‐2; DRS; IUS‐12	Study participants reported privacy concerns, enthusiasm, and low levels of distress.
42.	Ohneda et al. (2025)	Japan	Quantitative—longitudinal	112	33% (37) male 67% (75) female	41	PV carriers of BRCA 1/2 and other hereditary breast cancer‐related genes	WGS	Attending doctors, traiened by a clinical geneticist	Health care purposes	Pathogenic variants for hereditary cancers	In person	Immediately after, 12 months	Participants' comprehension level of the information; Psychological impact (general psychological distress); Cancer worry; Participants' attitudes toward addressing their genomic risks at the hospital; Hospital visiting behavior for medical surveillance; Sharing genetic information with relatives.	Kessler Psychological Distress Scale (K6), Cancer Worry Scale (CWS‐J) and a comprehension test.	While returning genetic risk information led to some positive health actions and comprehension was better among younger females, it had little significant impact on participants' mood or cancer worry.
43.	Abbott et al. (2025)	Scotland	Quantitative—survey	171	n.s.	n.s.	Rare conditions	WGS	n.s.	Health care purposes	Diagnostic status	Online	3–7 years	Personal utility and negative psychological outcomes	Personal Utility Scale (PrU) Feelings, About Genomic Testing Results (FACTOR).	The study found that both diagnosed and undiagnosed participants reported similar negative psychological outcomes on the FACTOR scale. However, specific feelings differed: undiagnosed individuals experienced more uncertainty, while diagnosed individuals reported higher levels of guilt.
44.	Nolan et al. (2024)	UK	Qualitative—semi‐structured interviews	32	50% (16) male 50% (16) female	24–75	Rare conditions or cancers	GS	Genetic counselors or medical geneticists	Health care purposes	Additional findings	Online or by telephone	29.4 (range 10.6–54) weeks	Comprehension and recollection of the AF's consent, along with psychological and behavioral reactions to the AF—such as family communication, satisfaction with the decision, and feelings of regret.	Semi‐structured interviews	Recipients AFs were often surprised and misunderstood their consent for these findings. While many ultimately valued the information, some, particularly younger women with BRCA AFs, struggled to conceptualize their disease risk and make management decisions.
45.	Yan et al. (2025)	SUA	Quantitative—survey	43	23.3% (10) male 76.7% (33) female	51.6	Health conditions having a suspected but unknown genetic etiology	WGS	Genetic counselors or medical geneticists	Health care purposes	Secondary genomic findings with limited to no medical actionability	In person or by telephone	2 weeks	Test‐related distress, Decision regret, Expected health anxiety, Perception of results as reassuring or troubling, Personal utility.	Multidimensional Impact of Cancer Risk Assessment (MICRA), Decision Regret Scale, a 5 points Likert scale, open questions	The study found low levels of distress, regret, and anxiety among participants receiving secondary genomic findings (LMA‐SFs).

*Note:* Assessment time point* mean duration from receipt of genomic sequencing results to assessment of their psychosocial impact. Sample size and assessment time points are reported as provided in the original studies.

Abbreviations: B‐IPQ = Brief Illness Perception Questionnaire; BAI = Beck Anxiety Inventory; BRFSS = Behavioral Risk Factor Surveillance System; CES‐D = Center for Epidemiologic Studies Depression Scale; DCS = Decisional Conflict Scale; DRS = Decision Regret Scale; EORTC‐QLQ‐C30 = European Organisation for Research and Treatment of Cancer Quality of Life Questionnaire‐C30; ES = exome sequencing; FACToR = Feelings About genomic Testing Results; GAD‐7 = Generalized Anxiety Disorder‐7; GHQ‐12 = General Health Questionnaire‐12; GS = genome sequencing; GSDS = Genetic Stigma Discrimination Scale; HADS = Hospital Anxiety and Depression Scale; HINTS = Health Information National Trends Survey; HLC = Health Locus of Control; IES = Impact of Event Scale; IQOLA = International Quality of Life Assessment; KOGS = Knowledge of Genetic Sequencing; LMA‐SFs = secondary findings with limited or no medical actionability; LOT‐R = Life Orientation Test‐Revised; NHANES = National Health and Nutrition Examination Survey; n.s = not specified; NS‐TADs = non‐syndromic thoracic aortic diseases; MICRA = Multidimensional Impact of Cancer Risk Assessment; PHQ‐9 = Patient Health Questionnaire‐9; PrU = Personal Utility Scale; PROMIS = Patient‐Reported Outcomes Measurement Information System; PUGS = Perceived Uncertainty of Genomic Sequencing; REALM = Rapid Estimate of Adult Literacy in Medicine; SF = secondary findings; STAI = State‐Trait Anxiety Inventory; SWD = Satisfaction With Decision; TMSI = Trait Meta‐Mood Scale; UNC‐GKS = University of North Carolina Genomic Knowledge Scale; VUS = variants of uncertain significance; WES = whole‐exome sequencing; WGS = whole‐genome sequencing.

Of the included studies, 27 have quantitative designs, 11 used qualitative designs and 7 adopted mixed‐methods approaches. Geographically, most studies were conducted in North America and Europe, with 36/45 in the USA (80%) and 8/45 in Europe (17.8%), 1/45 Japan (2.2%). Regarding study populations, non‐clinical samples (i.e., healthy adults) were included in 17 of 45 studies (37.8%), while clinical populations were examined in 28 of 45 studies (62.2%). The most frequently represented conditions were cancer (7/45, 15.5%), rare conditions (5/45, 11.1%), cardiac conditions (5/45, 11.1%), undiagnosed conditions (4/45, 8.8%) with other conditions represented less frequently. Sample sizes ranged from a minimum 10 participants (Hart et al. 2019) to a maximum of 1442 (Stuttgen et al. 2020). Participant ages ranged from 18 to 84 years, with most participants aged over 40 years (mean ages ranged between 46.5 to 63.9 years). Gender distribution was approximately balanced, with 3246 men (44.5%) and 4046 women (55.5%).

With respect to the type of testing, participants received WES results in 24/45 studies, and WGS results in 21/45 studies (46.7%). Regarding results disclosure, most studies reported that results were communicated by a professional with a genetics background (e.g., genetic counselors, medical geneticists), in 29 of 45 studies (64.4%). In the remaining studies (16/45, 35.6%) results were disclosed by specialist clinicians (e.g., cardiologists, oncologists, neurologists, endocrinologists) and researchers. In terms of the mode of delivery, genetic test results were most commonly delivered in person (26/45, 57.8%), followed by delivery via an online platform and/or email (15/45, 33.3%) and/or by telephone (8/45, 17.8%).

The most commonly reported motivation for pursuing WES/WGS testing was diagnostic or healthcare‐related purposes (38/45; 84.4%), followed by interest in genetic testing and research (12/45; 28.6%), and curiosity about one's genetic makeup (8/45; 19%) (Biesecker et al. 2018; Ormondroyd et al. 2020; Lewis et al. 2018; Zierhut et al. 2015; Zoltick et al. 2019). With respect to timepoints, the interval between receipt of results to assessment of psychosocial averaged between 3 and 6 months. The types of genetic testing results reported varied according to study population and research objective and were categorized as primary or incidental findings (12/45), negative (8/45), or positive results (6/45); carrier status or variant classification (pathogenic/likely pathogenic/non‐pathogenic/VUS) (10/45), and medically actionable or non‐actionable results (3/45).

The findings from quantitative and qualitative studies were analyzed separately, as these approaches capture different facets of the psychosocial outcomes through different methodologies. Quantitative studies primarily reported data derived from standardized surveys and validated instruments (e.g., MICRA, FACToR, GAD‐7, PHQ‐9, MHI‐5), whereas qualitative studies provided complementary insights that refined and deepened the understanding of participants' psychosocial responses.

### 3.2 Synthesis of Quantitative Studies

The synthesis of quantitative studies included 34 studies (27 quantitative and 7 mixed‐methods). The psychosocial outcomes identified were grouped into 3 categories, based on sentiment analysis of reported findings, polarity of the text and participant quotations: positive emotions (encompassing reactions and emotional states with positive valence, as well as statements indicating minimal or no perceived health implications), negative emotions and uncertainty (including negative emotional responses, perceptions of substantial health implications, and expressions of ambiguity). In addition, we also analyzed the social impact of the results, defined here as factors related to the sharing of genetic test results with family members, healthcare professionals and other relevant stakeholders.

#### 3.2.1 Positive Reactions

*Positive outcomes (e.g., positive experience, relief, empowerment)* were measured as outcomes in 16/34 studies, with medium to high levels of positive experiences consistently reported. Positive feelings in relation to genetic test results were more frequently reported 1 week after disclosure than 6 months post‐disclosure (this pattern likely reflects characteristics of the measurement scale; i.e., The Multidimensional Impact of Cancer Risk Assessment—MICRA) which assesses how often participants experienced specific emotions during the preceding week. Overall, the findings of the studies suggest positive short‐term responses to receiving health‐relevant genomic information, and alongside evidence of adequate adaptation to long‐term implications of results. Comparisons between participants receiving WES/WGS results and those receiving other medical outcomes such as family history report alone, type 2 diabetes or osteoporosis diagnoses (Christensen et al. 2021), revealed similar levels of positive affect across groups. However, across all post‐disclosure time points, individuals in the WGS/WES groups were more likely to report feelings of happiness, empowerment, and relief related to their test results (Beil et al. 2021; Christensen et al. 2021; Wynn et al. 2018).

*Quality of life* was assessed in 3/34 studies (Abbasciano et al. 2022; Sanderson et al. 2017; Sie et al. 2015). Across these studies, most participants reported being in excellent or good health at baseline, with no significant changes in quality of life measures observed in those who received negative WGS/WES results (Abbasciano et al. 2022); in contrast, participants with positive WGS/WES results, reported a decrease in quality of life at follow‐up (Sie et al. 2015).

*Satisfaction* with the decision to pursue WGS/WES was assessed in 8/34 studies, with most participants reporting high levels of satisfaction (Sie et al. 2015; Sanderson et al. 2017). Higher satisfaction was observed among participants who received genetic counseling compared to those who received results via web‐based platforms (Wynn et al. 2018; Biesecker et al. 2018). Overall, most participants described the testing experience as valuable, expressed satisfaction with the decision to undergo WGS/WES, and believed that the information obtained could be more useful in the future than at the time of disclosure (Sanderson et al. 2017). Many participants also indicated that they had gained new information that could be used to improve their health, felt a greater sense of control, and reported increased confidence in their ability to take action and plan for the future (Zoltick et al. 2019; Lewis et al. 2016). Nonetheless, participants often reported disappointment that their results did not provide more information and expressed unmet expectations regarding the potential benefits of testing (Zoltick et al. 2019; Roberts et al. 2018).

Another factor associated with more frequent feelings of well‐being and better overall psychological adjustment was the degree of *expectation fulfillment*, defined as the extent to which participants perceived alignment between their expectations of the genetic testing experience and the outcomes they derived from it (Griesemer et al. 2019).

#### 3.2.2 Negative Reactions and Uncertainty

*Anxiety* was assessed in 12/34 studies, with anxiety levels generally low at baseline and increased to moderate at least one of the post‐disclosure time points (typically at 6 weeks or 6 months), followed by a trend toward decreasing over time (Christensen et al. 2021; Sanderson et al. 2015; Sanderson et al. 2017; Gillman et al. 2022). Emotions related to future‐orientated concerns (e.g., anticipatory anxiety) were associated with increased anxiety immediately following results disclosure (as measured by the State–Trait Anxiety Inventory, STAI), as well as with greater distress and uncertainty on average within 1–6 months post‐disclosure (MICRA). Furthermore, the provision of genetic education or genetic counseling sessions alongside result disclosure was associated with lower levels of anxiety and test‐specific distress (V. van der Schoot et al. 2021).

An additional 5/34 studies examined *concerns* expressed by participants and found that such concerns were common but generally of low intensity (Roberts et al. 2018; Cheema et al. 2021; Zoltick et al. 2019; Zierhut et al. 2015; Skinner et al. 2017). Only a minority of participants expressed strong concerns, most frequently related to the confidentiality of results, potential financial or insurance implications, the limited clinical utility of findings for their specific condition, and the possibility of receiving unsolicited or unwanted information (Roberts et al. 2018). More commonly, participants reported moderate concerns, including uncertainty regarding the predictive accuracy of genomic results, challenges in interpreting genetic variants, concerns about the privacy of genetic data, and worries about potential implications for their children's health (Cheema et al. 2021; Zoltick et al. 2019). Overall, these concerns tended to diminish over time, with noticeable reductions observed within the weeks following the disclosure of sequencing results.

*Depressive symptoms* were assessed in 8/34 studies, and reported scores were generally low at baseline and moderate at one of the post‐disclosure time points. At follow up, mean depression scores decreased by 6 weeks and maintained stable at 6 months (Abbasciano et al. 2022; Vassy et al. 2017; Sapp et al. 2018). Depressive symptoms were assessed using standardized instruments, including the Depression subscale of the Hospital Anxiety and Depression Scale (HADS), the Patient Health Questionnaire (PHQ‐9), and the Center for Epidemiologic Studies Depression Scale (CES‐D).

*Test‐related distress* was measured in 18/34 studies and was generally reported to be low at both baseline and follow‐up. In a smaller subset of studies (Sanderson et al. 2015; Carrasco et al. 2023), elevated distress scores were observed at one or more post‐disclosure time points; however, these increases were transient and tended to return to baseline levels over time. In studies where result disclosure was accompanied by genetic education or genetic counseling, participants reported lower levels of distress (Rini et al. 2021; Lewis et al. 2016). Although the beneficial effects of genetic counseling appeared to attenuate within three to 6 months post‐disclosure, these short‐term improvements occurred during a particularly sensitive phase of the testing process and may therefore provide meaningful emotional relief for patients at the time of result communication.

*Decisional regret*, defined as distress or remorse associated with having made a particular decision, was assessed in 16/34 studies. Most participants reported little or no regret about undergoing genetic testing, with only a small proportion expressing regret or dissatisfaction related to their decision to undergo WSE/WSG (Roberts et al. 2018). When reported, dissatisfaction or regret was attributed to long waiting times before receiving test results. Additionally, greater regret was associated with inaction rather than action, with participants indicating that they would be more likely to regret not receiving genetic information than receiving it, even when results were uncertain or limited in clinical utility (Lawal et al. 2018).

*Uncertainty* following receipt of genetic test results was examined in 16/34 studies, and it was commonly reported as being low levels that remained stable over time. An exception was observed among participants who received secondary findings (10/18 studies) and who reported higher levels of uncertainty (Abbott et al. 2025; Umstead et al. 2020; Skinner et al. 2017). Notably, perceived accuracy of results decreased over time (particularly within 3 months post‐disclosure) as participants forgot much of the information received, whereas perceived ambiguity remained consistently low (Taber et al. 2018). The communication of genetic test results and individuals' understanding of their potential personal implications were found to significantly influence uncertainty related to recommended health behaviors, perception of result credibility, and overall psychosocial responses. Effective communication may therefore mitigate perceived health threats and provide substantial support during what is often perceived as a stressful period (Umstead et al. 2020).

#### 3.2.3 Social Impact

The extent to which genetic test results were shared with family members, healthcare professionals and other relevant stakeholders was measured in 9/34 studies. Participants most frequently communicated results with at least one first‐degree family member, such as a spouse or children (Lewis et al. 2016; Gillman et al. 2022; Miller et al. 2019) followed by communication with healthcare providers (Zoltick et al. 2019; Miller et al. 2019), and, less commonly, friends or acquaintances (Zierhut et al. 2015; Beil et al. 2021). Among participants who had not initially shared their results, a willingness or intention to do so in the future was often reported once they felt ready or when circumstances were considered more appropriate. Notably, the intention to disclose results was lower among individuals with positive results than in those who received negative results. However, while disclosure intentions among individuals who received positive results remained relatively stable over time, intentions increased over a 3‐month period for those with negative results it increases over time (Taber et al. 2018). Additionally, participants with negative results tended to cluster at two extremes—either being very likely or very unlikely to share results (Taber et al. 2018). Additionally, participants who shared their results reported high levels of family support during the genetic testing process and low levels of familial conflict. Behavioral lifestyle changes were reported infrequently; however, those who did report changes, health‐related behaviors such as modifications in diet and physical activity, changes in medication regimen, and seeking clinical evaluation and additional information, were perceived as more valuable than those who maintained their previous behaviors (Nolan et al. 2024; Zoltick et al. 2019).

### 3.3 Synthesis of Qualitative Data

The qualitative synthesis included data from 18 studies (11 qualitative, 7 mixed‐methods), with findings categorized using the same thematic framework applied to the quantitative data. With respect to methodology, qualitative studies were primarily collected through semi‐structured interviews (12/18), cross‐sectional interviews (2/18), or open‐ended questions.

*Positive reactions* were frequently reported (11/18 studies) by many participants. The value of the health information tended to outweigh the concerns or potential psychosocial burdens associated with managing genomic results. Participants who received negative genetic test results most often reported feelings of comfort, relief, and reassurance that their results were not indicative of a more serious condition (Beck et al. 2021; Cheema et al. 2021). In contrast, those who received positive results expressed gratitude for gaining additional insights into their health and for having an explanation or the opportunity to seek further medical guidance (Nolan et al. 2024; Taber et al. 2018; Lewis et al. 2016). These findings suggest that positive responses are not solely determined by the clinical nature of the result, but also by the extent to which genomic information reduces uncertainty, provides explanatory value, or supports future‐oriented decision‐making.

For participants with a pre‐existing, clinical diagnosed condition, receiving results often provided a sense of resolution or closure. When results had the potential or a definitive explanatory role in relation to their existing condition, participants reported feelings of validation and described being able to conclude the search for answers. Participants attributed their satisfaction to an increased ability to take a more proactive role managing health, as well as to being able to attribute their symptoms to a diagnosis (Skinner et al. 2017; Smith et al. 2022).

Participants valued the information received for its potential in disease prevention and early detection, both for themselves and for family members (Lewis et al. 2016). The information was perceived as personally useful, contributing to increased vigilance and self‐awareness regarding health‐related choicesaa (Schoot et al. 2021). Additionally, participants reported satisfaction in contributing to future research and advancing knowledge, describing the testing process as interesting and, at times, fulfilling their personal curiosity (Peter et al. 2022; Cheema et al. 2021).

Studies indicate that emotional responses tend to become more positive over time as the participants have had the opportunity to adjust to the information received (Nolan et al. 2024). Family history also appeared to play a reassuring role: participants with a known family history reported feeling more prepared to manage their results, whereas those with an uninformative family history often minimized the risks due to the absence of affected family members. Age was identified as an additional factor influencing positive affects; participants over 40 years of age (mean ages ranging from 46.5 to 63.9 years) frequently reported that the absence of disease to date reduced their perceived likelihood of future ones, or that other health concerns warranted more immediate attention (Nolan et al. 2024; Lewis et al. 2016). Furthermore, effective coping skills and a good understanding of genetic information and its health implications also contributed to increased positive emotional responses (Nolan et al. 2024). Positive adaptation is influenced not only by the content of the results themselves, but also by individuals' pre‐existing beliefs, coping resources, health experiences, and ability to integrate genomic information into their broader life context.

*Neutral or ambivalent reactions* were found in 6/18 studies. Negative genetic test results often elicited little emotional response and were perceived as unremarkable or neutral results, and in some cases reassuring, with participants reporting no reason for concern given the absence of anything problematic (Beck et al. 2021). However, some participants with negative genetic test results reported feelings of disappointment, reflecting unmet expectations about their health status (Peter et al. 2022; Beck et al. 2021). Ambivalent responses were also reported by some participants who appreciated the potential health benefits of genomic information while simultaneously expressing concern about associated risks (Lewis et al. 2016). The coexistence of reassurance and disappointment highlights that psychosocial responses to genomic testing are often shaped by expectations, with individuals evaluating results not only in terms of medical significance but also in relation to their anticipated informational and emotional benefits.

*Negative reactions* were less common; in 7/18 studies, participants expressed negative feelings, including concern, disbelief, disappointment, confusion, frustration or the feelings of unpreparedness for receiving such results. Concerns were often linked to potential implications for their physical and mental health, as well as for that of their families (Lewis et al. 2016; Sanderson et al. 2017). Participants also raised concerns regarding financial costs and health insurance implications (Cheema et al. 2021). Some participants described disappointment stemming from unmet expectations, reporting that they had anticipated more comprehensive or personally relevant information (Peter et al. 2022; Smith et al. 2022). Participants also reported feelings of surprise when the results provided them with information that was unexpected or for which they felt they had little or no prior preparation (Nolan et al. 2024). Disbelief, either in the validity of the results or in the testing process itself, was rarely reported and occurred primarily among participants who sought a definitive diagnosis through testing but did not receive one, often leading to feelings of frustration or disappointment (Skinner et al. 2017). In contrast, the identification of variants associated with rare diseases elicited intense negative emotional responses, including heightened stress, substantial worry, and the perception of having learned profoundly impactful information (Abbott et al. 2025). Despite these reactions, participants generally expressed high levels of trust in the genetic testing process. Many described feelings of reassurance, emphasizing that their testing involved the most advanced technology available. For some, WGS/WES represented a form of “final hurrah” in their diagnostic journey—a hopeful attempt to find answers after years of uncertainty (Skinner et al. 2017). Importantly, negative reactions appeared to arise less from the genomic information itself and more from unmet expectations, difficulties interpreting uncertainty, or the perceived consequences of the findings.

#### 3.3.1 Uncertainty

In 6/18 studies participants reported having unresolved questions or experiencing confusion after receiving their results. These uncertainties were subsequently addressed through genetic counseling sessions, consultations with physician, online resources or discussions with relatives (Beck et al. 2021; Jamal et al. 2024). Participants who received their results in a genetic counseling session reported significantly fewer residual questions compared to those who received their results through other means (Umstead et al. 2020). Feelings of confusion were primarily attributed to difficulties in understanding complex genetics concepts, as well as challenges in interpreting probabilistic and multifactorial causation in genetics (Beck et al. 2021; Peter et al. 2022); uncertainty most commonly related to the prognosis, the likelihood of obtaining a definitive diagnosis, and the medical actionability of the results (Umstead et al. 2020).

#### 3.3.2 Social Impact

Several studies (6/18) reported that participants shared their results with family members, friends and/or healthcare provider. Most participants discussed their results with family and/or friends, either to convey potential health risks to relatives, or because they found the information interesting or sought support in interpreting the findings (Lewis et al. 2016). Participants also expressed concerns about implications of results for family implications (e.g., how the condition would manifest in relatives, whether they would should undergo genetic testing, and which relatives they should be informed about their results) (Nolan et al. 2024). Concerns were also related to the well‐being of the family; participants reported that genetic information could be distressing or difficult for some family members (e.g., young children), and therefore chose to communicate results selectively (Nolan et al. 2024). Another factor that influenced disclosure of results was whether communication was perceived as an obligation/responsibility: when disclosure was framed as a responsibility, results were more frequently shared, whereas framing disclosure as an individual choice was associated with more selective communication (Lewis et al. 2016). Disclosure was most often experienced as a responsibility when results had implications for children, and results that were clear and easy to understand were more likely to be shared with family members (Lewis et al. 2016). Participants also reported sharing genetic information with their healthcare providers, or expressed intentions to do so; however, some participants did not think that the results would affect their care or perceived the information not immediately relevant (Lewis et al. 2016; Lawal et al. 2018). When participants received support from the healthcare providers, their understanding of the health implications improved; this, in turn, facilitated more meaningful conversations with clinicians and fostered a stronger sense of solid support (Lewis et al. 2016; Shaibi et al. 2020). Collectively, these findings indicate that communication is not merely a consequence of receiving genomic results but a central mechanism through which individuals process information, negotiate responsibility, seek support, and construct meaning around the implications of genomic risk for themselves and their families.

Overall, carrier status findings were more commonly associated with communication and family disclosure outcomes, with participants frequently sharing results with relatives because of their reproductive implications and perceived familial utility (Biesecker et al. 2018; Reid et al. 2020; Gillman et al. 2022). In contrast, actionable findings, particularly those related to cancer predisposition and rare diseases, were more frequently associated with emotional responses, distress, uncertainty, and complex decision‐making processes (Carrasco et al. 2023; Bradbury et al. 2018; Nolan et al. 2024; Abbott et al. 2025). Variants of uncertain significance were predominantly linked to cognitive processes related to uncertainty management, interpretation of risk, and adaptation to incomplete information (Lawal et al. 2018; Skinner et al. 2017).

The integration of quantitative and qualitative findings suggests that psychosocial responses to WES/WGS are dynamic processes shaped by individuals' interpretation of genomic information rather than by the objective nature of the results alone. Quantitative studies consistently reported low levels of long‐term anxiety, depression, distress, uncertainty, and decisional regret (Abbasciano et al. 2022; Vassy et al. 2017; Sapp et al. 2018; Roberts et al. 2018), while qualitative findings helped explain these patterns by showing how participants actively engaged in meaning‐making, reframed uncertainty, and incorporated genomic information into their existing health narratives (Skinner et al. 2017; Smith et al. 2022; Nolan et al. 2024). For many participants, uncertainty was not experienced solely as a source of distress but also as an opportunity for future clarification, allowing them to view inconclusive or negative results as part of an ongoing diagnostic journey rather than as definitive endpoints (Skinner et al. 2017; Beck et al. 2021; Jamal et al. 2024). Similarly, the high levels of satisfaction and empowerment reported quantitatively (Sanderson et al. 2017; Wynn et al. 2018; Biesecker et al. 2018; Zoltick et al. 2019) were mirrored by qualitative accounts describing increased perceived control, validation of lived experiences, and the ability to make informed health and life‐planning decisions (Skinner et al. 2017; Smith et al. 2022; Lewis et al. 2016). Social outcomes also appeared to function as important mediators of psychosocial adaptation. Quantitative studies documented frequent result disclosure and high levels of family support (Lewis et al. 2016; Gillman et al. 2022; Miller et al. 2019), whereas qualitative studies demonstrated that communication with relatives and healthcare professionals facilitated understanding, reduced uncertainty, reinforced coping strategies, and contributed to the development of a shared framework for interpreting genomic information (Lewis et al. 2016; Umstead et al. 2020; Shaibi et al. 2020). Together, these findings suggest that the psychosocial impact of genomic sequencing is best understood as an interactive process in which emotional, cognitive, behavioral, and social responses continuously influence one another over time.

**FIGURE 2:**
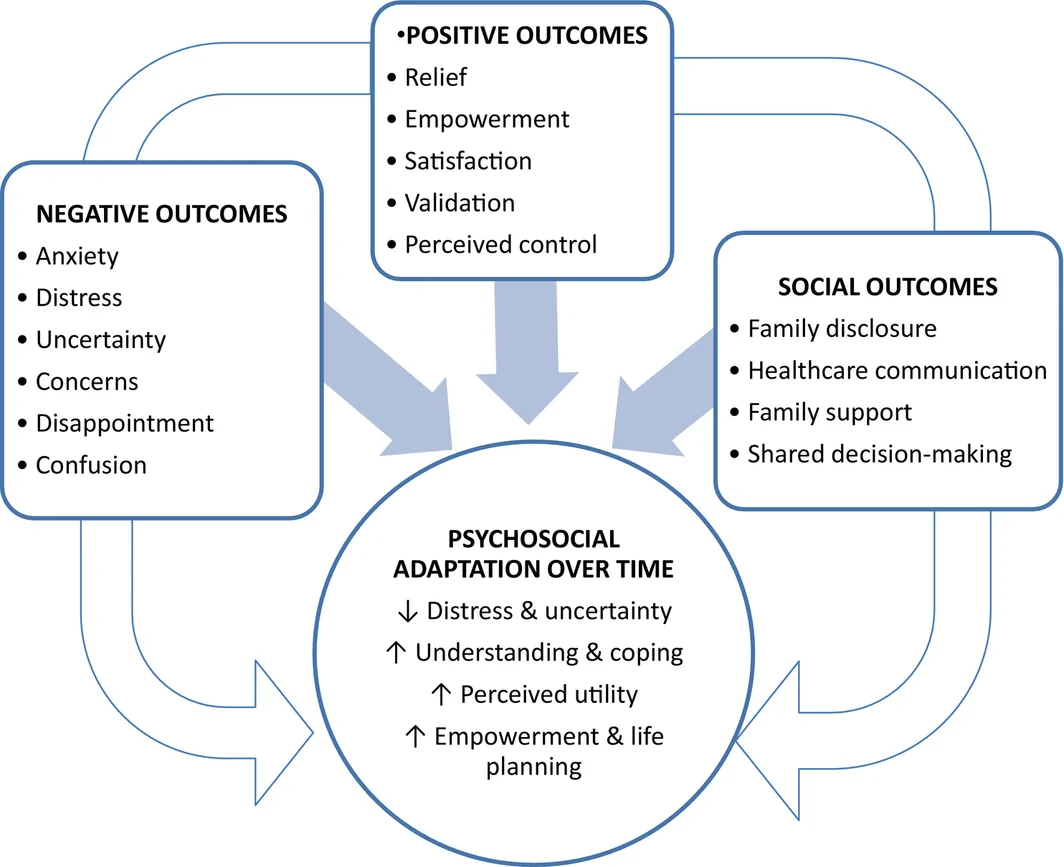
**Psychosocial** **outcomes following the receipt of genomic sequencing results**. Main outcomes are grouped into negative (e.g., anxiety, distress, uncertainty), positive (e.g., relief, empowerment, satisfaction), and social outcomes (e.g., family disclosure, healthcare communication, support). These outcomes may influence psychosocial adaptation over time, reflected in reduced distress and uncertainty and increased understanding, coping, perceived utility, and empowerment. Arrows indicate dynamic relationships between outcomes and adaptation.

The methodological quality of the included studies was assessed using the Mixed Methods Appraisal Tool (Hong et al. 2018). Most studies met the majority of the appraisal criteria and were judged to be of moderate methodological quality. Randomized controlled trials generally demonstrated the lowest risk of bias, whereas observational studies were mainly limited by sample representativeness, self‐selection bias, and attrition. Qualitative studies showed appropriate methodological congruence, although reflexivity and researcher influence were not consistently reported. Figure 2 provides an overview of the psychosocial factors identified across studies; positive responses such as relief and empowerment are interconnected with negative outcomes such as anxiety and uncertainty, and with social outcomes such as family and healthcare communication.

## 4 Discussion

The present paper synthesizes findings from 45 studies examining psychosocial responses to WGS/WES results. Overall, participants most frequently reported positive reactions, such as relief, reassurance, and empowerment, whereas negative emotions, such as anxiety, distress, and uncertainty were generally mild and tended to decrease over time, particularly when results disclosure was accompanied by counseling. Neutral or ambivalent responses were most commonly observed following uninformative or negative results. Genomic testing also influenced social interactions, with participants selectively sharing results with family members and healthcare providers. Collectively, these findings suggest that receiving WGS/WES results is largely manageable and perceived as valuable, with predominantly short‐term negative effects and more enduring informational and social benefits.

The integration of quantitative and qualitative findings revealed a largely consistent pattern across methodologies. Quantitative studies showed that most participants experienced low levels of long‐term anxiety, depression, distress, uncertainty, and decisional regret, while qualitative studies provided insight into the processes underlying these outcomes (Abbasciano et al. 2022; Vassy et al. 2017; Sanderson et al. 2017; Roberts et al. 2018; Lawal et al. 2018; Umstead et al. 2020). Specifically, qualitative findings demonstrated that participants actively interpreted, reframed, and incorporated genomic information into their personal health narratives, often transforming initial uncertainty into a sense of control, preparedness, or future opportunity (Lewis et al. 2016; Skinner et al. 2017; Beck et al. 2021; Peter et al. 2022; Nolan et al. 2024). Both evidence streams therefore suggest that psychosocial adaptation is influenced less by the objective nature of genomic results and more by how individuals understand and integrate those results into their broader life context (Lewis et al. 2016; Schoot et al. 2021; Skinner et al. 2017; Peter et al. 2022).

Although qualitative studies captured a wider range of nuanced emotional responses, including ambivalence, identity‐related concerns, and meaning‐making processes, both quantitative and qualitative evidence converged in showing that positive or adaptive responses generally outweighed sustained negative psychological outcomes (Griesemer et al. 2019; Nolan et al. 2024; Lewis et al. 2016; Peter et al. 2022).

Our findings are consistent with the broader literature, which indicates that, for most individuals, emotional distress associated with receiving WGS/WES genetic test results is modest (Vassy et al. 2017; Robinson et al. 2019). Although overall levels of emotional distress were generally low, some variability in psychological responses was observed depending on the type of result received. In particular, participants who received positive genetic test results reported fewer positive responses, suggesting a potential need for enhanced emotional support in this subgroup (Niu et al. 2019). Several studies (Umstead et al. 2020; Rego et al. 2019) reported low levels of distress among healthy adults, which may reflect a self‐selection of participants who pursue genomic testing out of curiosity to explore their genetic makeup and only undergo testing if they anticipate that they will be able to cope well with the results. Another explanation for the relatively low levels of distress is the limited clinical significance of many results; for many adult participants, the test results did not lead to immediate health implications or actionable interventions. Additionally, reports of low distress within this population may reflect a sampling bias, as participants from clinical populations, who may experience higher levels of distress, are often underrepresented in research due to illness related barriers (Robinson et al. 2019). Receipt of VUSs was associated with higher uncertainty and more frequent testing related concerns. In this context, Mighton et al. (2021) highlighted the critical role of healthcare providers in supporting participants' coping strategies and psychosocial responses to uncertain genomic results.

This review deliberately integrated both quantitative and qualitative research methodologies to capture a broader spectrum of data and to address inherent limitations of each approach. Quantitative studies reported data collected using standardized instruments (e.g., MICRA, HADS, FACToR, GAD‐7), enabling replicability and statistical analyses that facilitate comparisons across different settings or testing contexts (Field 2013). Qualitative studies (e.g., interviews, focus groups) complemented these standardized measures by offering more nuanced, multidimensional insights into participants' experiences by capturing issues that may be less readily quantified, such as uncertainty arising from inconclusive results and concerns about the familial implications of genomic findings (Payne et al. 2008; Li et al. 2019). By exploring how individuals interpret and make meaning of their results, qualitative methods provided a richer perspective on the psychosocial impact of genomic testing.

Disappointment emerged as a salient negative response associated with WGS/WES testing, underscoring the importance of appropriately framing the potential benefits and limitations of genomic testing. Expectations of actionable or personally meaningful outcomes may be shaped by broader societal narratives surrounding genomics and by the complexity of the consent process itself. These findings highlight the need for careful expectation management as a core component of pre‐ and post‐test communication, in order to support more realistic appraisals of genomic information and reduce the likelihood of unmet expectations. Overall, the predominance of positive responses highlights the perceived value of genomic information for many individuals. Positive psychosocial outcomes appear to be shaped by a combination of personal, contextual, and procedural factors, including individuals' health status and life stage, the passage of time and subsequent adaptation to results, family history, and the manner in which results are communicated. Together, these factors underscore the importance of context‐sensitive result disclosure and ongoing support to maximize the potential benefits of WGS/WES testing. Decisions about sharing genomic results with family members were shaped by whether disclosure was perceived as a personal choice or a moral obligation. When results had potential clinical implications for relatives, disclosure was more likely, whereas non‐disclosure was most often motivated by concerns about protecting family members' well‐being. These patterns highlight the relational and ethical dimensions of genomic information and underscore the need for guidance that supports individuals in navigating family communication decisions.

Although family communication emerged as an important theme across studies, the available evidence did not allow robust comparisons between countries or healthcare systems. Most studies originated from the United States and other high‐income Western settings, limiting the ability to determine whether disclosure practices are influenced by cultural norms, healthcare structures, or differences in access to genetic services.

## 5 Implications

Overall, our findings align with previous evidence suggesting that WGE/WES do not generally result in negative psychosocial outcomes and are often perceived in ways that extend beyond traditional notions of clinical utility, encompassing broader personal and social implications (Foster et al. 2009; Turrini and Prainsack 2016). Rather than being experienced as a single diagnostic event, WHS/WES appear to function as a valuable healthcare resource, that individuals may interpret, revisit and utilize throughout a person's life. This dynamic use of genomic information is reinforced by continuous advances in genomic technologies which allow results to be reinterpreted and reclassified as scientific knowledge evolves, thereby altering both the availability and perceived significance of results over time.

From a clinical perspective, these findings underscore the importance of embedding WGS/WES within a longitudinal model of care rather than treating testing as a one‐off intervention. In particular, the review highlights the value of providing both pre‐test and post‐test genetic counseling. Pre‐test education that clearly communicates the range of possible results, their limitations, and their potential personal and familial implications may help align expectations and reduce later disappointment. Post‐test counseling plays a critical role in supporting patients' understanding of results, contextualizing uncertainty, and facilitating adaptive coping and informed decision‐making. Evidence suggests that such counseling is associated with more positive emotional responses, improved communication with healthcare providers, and greater perceived benefit from testing (Christensen et al. 2021; Rini et al. 2021; Lewis et al. 2018). In addition, some individuals may benefit from targeted psychological support or referral to mental health services, particularly when receiving highly actionable findings, secondary findings, or results associated with significant uncertainty or emotional burden. Together, these findings suggest that effective integration of WGS/WES into clinical practice requires not only technical expertise but also sustained psychosocial support, clear communication strategies, and opportunities for re‐engagement with results as their meaning evolves over time. However, the implementation of comprehensive support pathways may be constrained by the limited availability of genetic counseling services and the increasing demand for genomic testing.

From a research perspective, the review highlights the need for more sensitive measurement instruments to capture more accurately the range and nuance of emotional reactions. Advancing psychosocial impact assessment in this field will require continued development and refinement of instruments that assess test‐specific distress, perceived uncertainty, and positive responses to testing (Li et al. 2019). Existing validated instruments such as MICRA (Cella et al. 2002) and FACToR (Li et al. 2019) illustrate ongoing efforts to move beyond traditional clinical endpoints by capturing the complex emotional and social dimensions of genomic testing. Future research would benefit from broader adoption, adaptation, and validation of such tools across diverse populations and testing contexts, enabling more precise comparisons and a deeper understanding of individual differences in psychosocial outcomes.

## 6 Limitations

This review has several limitations. A primary limitation arises from the heterogeneity of the included studies which constrained direct comparisons of psychosocial outcomes across studies. Sources of variability included differences in study design, clinical contexts (e.g., diagnostic versus healthy cohorts), varying time periods of outcome assessments (ranging from immediately post‐disclosure to up to 3 years later) and the instruments used to measure psychosocial impact. For instance, some studies used general psychological scales such as HADS to assess anxiety and depression, while others focused on test‐specific or anticipatory emotional responses related to potential genetic results. Additionally, the review synthesized findings from participants who received a wide range of WGS/WES results across both clinical or research settings; therefore, caution is warranted when generalizing the findings, as psychosocial responses are likely to differ substantially according to individuals' health status, motivation for testing, type of result received, healthcare context in which testing occurred.

A further limitation relates to the instruments used to assess the psychological impact. Measures were frequently adapted to suit specific study contexts, varied considerably across studies, and were not always sensitive to subtle or nuanced changes in psychosocial responses. Additionally, the inconsistencies in data reporting across the studies occasionally limited the extraction of all relevant information required for the data synthesis. Finally, in terms of diversity, the studies included had predominantly white, highly educated, English‐speaking participants with a high socioeconomic status, limiting the generalizability of the findings. Future research would benefit from the inclusion of more diverse populations to better capture the full range of psychological responses to WGS/WES across different cultural and socioeconomic contexts.

Another important limitation is the geographical concentration of the evidence. Most included studies were conducted in Western, high‐income countries, particularly the United States. Consequently, the findings may not fully reflect psychosocial experiences in culturally diverse populations or healthcare systems with different models of genetic service delivery. As genomic medicine increasingly seeks to improve representation and genetic diversity in clinical research, a similar effort is needed in psychosocial genomics research to ensure that the social, cultural, and emotional implications of genomic testing are understood across diverse populations.

## 7 Conclusions

Receipt of genomic sequencing results can elicit a broad range of emotional responses, from surprise and confusion to relief and satisfaction. This review suggests that, although initial emotional challenges may occur, most people well over time. Disclosure of results to relatives was common and appeared to be closely linked to perceived health implications of the findings. Importantly, result disclosure accompanied by genetic counseling was associated with reduced distress, underscoring its value during what can be a psychologically demanding phase of care. These findings support the integration of collaborative care models in genomic medicine, in which physicians and mental health professionals work together to provide comprehensive, person‐cantered support. Such collaboration can help individuals contextualize genetic information within their personal and familial circumstances, manage uncertainty, and address psychosocial concerns as they arise.

## Author Contributions

**Monica Albu:** conceptualization, methodology, data curation, formal analysis, investigation, project administration, screening and study selection, writing – review and editing, writing – original draft. **Andrada Ciucă:** conceptualization, methodology, data curation, investigation, screening and study selection, writing – review and editing. **Ramona Moldovan:** conceptualization, methodology, data curation, investigation, screening and study selection, writing – review and editing. All authors read and approved the final manuscript.

## Funding

The work of Andrada Ciucă was supported by the European Union's Horizon 2020 Research and Innovation Program (945151). The work of Ramona Moldovan was supported by the European Union's Horizon 2020 Research and Innovation Program (945151), Manchester NIHR BRC (NIHR203308), and MRC MR/Y008383/1.

## Disclosure

*AI statement*: The authors did not use AI or AI‐assisted technologies during the preparation of this manuscript.

## Ethics Statement

Ethical approval was not required as the article analyzes data publicly presented in other published articles.

## Consent

The authors have nothing to report.

## Conflicts of Interest

The authors declare no conflicts of interest.

## Supporting information


**Data S1:** PRISMA 2020 checklist.

**Data S2:** jgc470291‐sup‐0002‐Supinfo2.docx.

## Data Availability

The datasets generated during and/or analyzed during the current study are available from the corresponding author on reasonable request.
