# Supplementary material for: The Psychosocial Impact of Receiving Whole Genome and Whole Exome Sequencing Results in Adults: A Systematic Review
Source: J Genet Couns. 2026 Sep 24;35(5):e70291. doi: 10.1002/jgc4.70291 (PMC13602446; doi:10.1002/jgc4.70291)
Supplement: Supplementary file 1 [file JGC4-35-0-s001.docx]

**MMAT criteria**

**For qualitative studies**

C1 – The qualitative approach is appropriate to answer the research question.

C2 – The data collection methods are adequate to address the research question.

C3 – The findings are adequately derived from the data.

C4 – The interpretations are sufficiently substantiated by the data.

C5 – There is coherence between the research question, methodology, data collection, analysis, and interpretation.

**For quantitative descriptive / observational studies**

C1 – The sampling strategy is relevant to address the research question.

C2 – The sample is representative of the target population.

C3 – The measurements are appropriate and valid.

C4 – There is a low risk of non-response bias and/or confounding.

C5 – The statistical analysis is appropriate to answer the research question.

**For RCT**

C1 – Randomization was appropriately performed.

C2 – Groups were comparable at baseline.

C3 – Outcome data were complete.

C4 – Outcome assessment was conducted appropriately.

C5 – Participants adhered adequately to the assigned intervention.

| **Overall Rating** | **Interpretation** |
| --- | --- |
| **Low** | All 5 criteria fulfilled (5/5) |
| **Low–Moderate** | 4–5 criteria fulfilled, with some insufficient reporting |
| **Moderate** | 3–4 criteria fulfilled |
| **Moderate–High** | 2–3 criteria fulfilled |
| **High** | ≤2 criteria fulfilled |

| **No.** | **Study** | **Design** | **Overall rating** |
| --- | --- | --- | --- |
| 1. | Abbasciano et al., 2022 | Quantitative – Prospective and non-interventional feasibility study | Moderate |
| 2. | Beck et al., 2021 | Qualitative – Semi-structured interviews | Moderate |
| 3. | Beil et al., 2021 | Quantitative – Observational | Moderate |
| 4. | Biesecker et al., 2018 | Quantitative – Randomized Clinical Trial | Low–Moderate |
| 5. | Butterfield et al., 2019 | Quantitative – Correlational descriptive | Moderate |
| 6. | Carrasco et al., 2022 | Quantitative – Randomized Clinical Trial | Low–Moderate |
| 7. | Cheema et al., 2021 | Qualitative – Semi-structured interviews | Moderate |
| 8. | Christensen et al., 2021 | Quantitative – Randomized Clinical Trial | Low–Moderate |
| 9. | Ormondroyd et al., 2020 | Qualitative – Semi-structured interviews | Moderate |
| 10. | Gillman et al., 2021 | Quantitative – Longitudinal prospective | Moderate |
| 11. | Griesemer et al., 2019 | Quantitative – Descriptive | Moderate |
| 12. | Hart et al., 2015 | Qualitative – Semi-structured interviews | Moderate |
| 13. | Jamal et al., 2023 | Qualitative – Cross-sectional interviews | Moderate |
| 14. | Lawal et al., 2018 | Mixed methods – Experimental | Moderate |
| 15. | Lewis et al., 2015 | Mixed methods – Exploratory | Moderate |
| 16. | Lewis et al., 2018 | Quantitative – Randomized Controlled Trial | Low |
| 17. | Miller et al., 2019 | Quantitative – Descriptive | Moderate |
| 18. | Niu et al., 2019 | Quantitative – Randomized Controlled Trial | Low |
| 19. | Peter et al., 2022 | Mixed methods – Survey and in-depth interviews | Moderate |
| 20. | Reid et al., 2020 | Quantitative – Exploratory | Moderate |
| 21. | Rini et al., 2017 | Quantitative – Correlational | Moderate |
| 22. | Rini et al., 2019 | Quantitative – Longitudinal | Moderate |
| 23. | Rini et al., 2021 | Quantitative – Randomized Controlled Trial | Low |
| 24. | Roberts et al., 2018 | Quantitative – Prospective | Moderate |
| 25. | Roche et al., 2018 | Quantitative – Correlational | Moderate |
| 26. | Sanderson et al., 2015 | Mixed methods – Observational and interview | Moderate |
| 27. | Sanderson et al., 2017 | Mixed methods – Exploratory longitudinal | Moderate |
| 28. | Sapp et al., 2018 | Quantitative – Exploratory | Moderate |
| 29. | Shaibi et al., 2020 | Qualitative – Descriptive | Moderate |
| 30. | Sie et al., 2015 | Quantitative – Observational prospective | Moderate |
| 31. | Skinner et al., 2016 | Qualitative – Ethnographic observations and semi-structured interviews | Moderate |
| 32. | Skinner et al., 2017 | Qualitative – Ethnographic observations and semi-structured interviews | Moderate |
| 33. | Smith et al., 2022 | Qualitative – Semi-structured interviews | Moderate |
| 34. | Stuttgen et al., 2020 | Quantitative – Survey | Moderate |
| 35. | Taber et al., 2017 | Mixed methods – Experimental longitudinal | Moderate |
| 36. | Umstead et al., 2019 | Mixed methods – Randomized Controlled Trial | Moderate–High |
| 37. | van der Schoot et al., 2021 | Qualitative – Semi-structured interviews | Moderate |
| 38. | Vassy et al., 2017 | Quantitative – Randomized Trial | Low |
| 39. | Wynn et al., 2018 | Quantitative – Longitudinal | Moderate |
| 40. | Zierhut et al., 2015 | Quantitative – Survey | Moderate–High |
| 41. | Zoltick et al., 2019 | Quantitative – Prospective longitudinal | Moderate |
| 42. | Ohneda et al., 2025 | Quantitative – Longitudinal | Moderate |
| 43. | Abbott et al., 2025 | Quantitative – Survey | Moderate |
| 44. | Nolan et al., 2024 | Qualitative – Semi-structured interviews | Moderate |
| 45. | Yan et al., 2025 | Quantitative – Survey | Moderate |
